# Sustained efficacy of acupuncture in post-stroke dysphagia: a systematic review and meta-analysis

**DOI:** 10.3389/fneur.2026.1889347

**Published:** 2026-09-21

**Authors:** Zixuan Liao, Kailin Xu, Ling Gao, Miaosen Huang, Haipeng Jin

**Affiliations:** 1 College of Acupuncture-Moxibustion and Tuina, Fujian University of Traditional Chinese Medicine, Fuzhou, China; 2 Xiamen TCM Hospital Affiliated to Fujian University of Traditional Chinese Medicine, Xiamen, China

**Keywords:** acupuncture, dysphagia, long-term efficacy, meta-analysis, randomized controlled trial, stroke, sustained efficacy, systematic review

## Abstract

**Objective:**

To systematically evaluate the short-term and long-term (≥3 months post-treatment) efficacy and safety of acupuncture for post-stroke dysphagia (PSD), providing comprehensive evidence for clinical rehabilitation strategies.

**Methods:**

Conducted in strict adherence to the PRISMA guidelines, a comprehensive search of Web of Science, PubMed, Embase, the Cochrane Library, VIP, Wanfang, CNKI, and CBM was performed from inception to March 18, 2026 for randomized controlled trials (RCTs) published in English or Chinese. Two independent reviewers performed study selection, data extraction, and risk of bias assessment. Meta-analysis was conducted utilizing Review Manager 5.4 and Stata 18.0.

**Results:**

A total of 23 RCTs involving 2,224 patients were included. For short-term outcomes, acupuncture interventions significantly improved the Water Swallowing Test (WST) effective rate (RR = 1.50, 95% CI [1.31, 1.73], *p* < 0.001) and reduced Standardized Swallowing Assessment (SSA) scores (MD = −3.33, 95% CI [−4.46, −2.19], *p* < 0.001). These significant clinical benefits were robustly sustained for long-term outcomes (≥ 3 months follow-up), as evidenced by the WST effective rate (RR = 1.38, 95% CI [1.21, 1.58], *p* < 0.001), objective SSA scores (MD = −4.92, 95% CI [−6.54, −3.31], *p* < 0.001), and subjective SWAL-QOL scores (MD = 23.56, *p* = 0.010). Subgroup analyses indicated that initiating acupuncture during the subacute/recovery phase yielded pronounced WST improvements. Regarding stimulation modalities, while electroacupuncture showed a marginal potential advantage for the WST (*p* = 0.08), no statistically significant superiority was established for the objective SSA scores (*p* = 0.56). Acupuncture-related adverse events were confined to mild local reactions (RD = 0.11, *p* < 0.001).

**Conclusion:**

Acupuncture is a safe and potentially effective approach for post-stroke dysphagia, improving both short-term swallowing function and quality of life. Notably, these benefits appear to be sustained for at least 3 months post-treatment. Nevertheless, the overall certainty of evidence for short-term efficacy ranges from low to moderate, and the certainty for long-term sustained efficacy ranges from low (WST and SSA) to very low (SWAL-QOL), primarily due to inherent performance bias, publication bias, and imprecision due to limited long-term sample sizes, rather than clinical inconsistency.

**Systematic review registration:**

https://www.crd.york.ac.uk/PROSPERO/view/CRD420261345975, identifier (CRD420261345975).

## 1 Introduction

Post-stroke dysphagia (PSD) is one of the most common and debilitating complications following an acute cerebrovascular event, with an incidence exceeding 50% in acute stroke patients (1). Impaired swallowing function not only severely compromises patients’ nutritional status and quality of life but also significantly increases the risk of aspiration pneumonia, malnutrition, and dehydration, which collectively remain the leading causes of non-neurological mortality in stroke survivors (2–4). Consequently, restoring safe and effective swallowing function is a paramount priority in the stroke rehabilitation trajectory.

Currently, conventional swallowing rehabilitation therapy (CSRT) serves as the first-line clinical intervention for PSD. This therapy typically encompasses dietary modifications, compensatory maneuvers, and neuromuscular exercises (5). However, the real-world clinical outcomes of CSRT are frequently constrained by prolonged treatment durations, poor patient compliance, and the inevitable “rehabilitation plateau” (6). More importantly, a significant proportion of patients with moderate-to-severe dysphagia struggle to maintain the therapeutic benefits once the intensive training period ceases. This highlights the urgent need for complementary neuromodulatory interventions that can facilitate sustained functional recovery (5, 6).

Acupuncture, a cornerstone of traditional East Asian medicine, has been increasingly integrated into stroke rehabilitation guidelines worldwide, including the recent European Stroke Organisation consensus, due to its minimal invasiveness and promising neuromodulatory effects (1). Accumulating neurobiological evidence suggests that targeted acupuncture stimulation, such as electroacupuncture, can accelerate the reorganization of the swallowing motor cortex, upregulate brain-derived neurotrophic factor (BDNF) expression, and reactivate the impaired peripheral-central swallowing reflex arc (7, 8). Despite a growing body of randomized controlled trials (RCTs) demonstrating the efficacy of acupuncture in treating PSD, the majority of existing systematic reviews and meta-analyses have exclusively focused on the immediate, short-term outcomes measured at the end of the intervention period (9, 10).

The sustainability of these clinical benefits remains largely unexplored. Specifically, it is unclear whether the neuroplasticity induced by acupuncture translates into durable, long-term functional improvement evaluated at 3 months or more post-treatment. To bridge this critical knowledge gap, we conducted this comprehensive systematic review and meta-analysis. By rigorously synthesizing the latest high-quality RCTs, this study aims to systematically evaluate and compare the short-term and long-term efficacy and safety of acupuncture for PSD. Ultimately, we seek to determine the sustained therapeutic value of acupuncture, thereby providing robust, evidence-based guidance for optimizing full-cycle, long-term rehabilitation strategies for stroke survivors.

## 2 Materials and methods

This systematic review and meta-analysis was conducted in strict accordance with the Preferred Reporting Items for Systematic Reviews and Meta-Analyses (PRISMA) statement (11). The research protocol was prospectively registered in the International Prospective Register of Systematic Reviews (PROSPERO) under the registration number CRD420261345975 [available at: https://www.crd.york.ac.uk/PROSPERO/view/CRD420261345975, (12)].

### 2.1 Literature search strategy

A comprehensive and systematic literature search was conducted across both English and Chinese electronic databases from their respective inceptions to March 18, 2026. The searched English databases included PubMed, Embase, the Cochrane Library, and the Web of Science Core Collection, with no journal-tier restrictions. The Chinese databases encompassed the China National Knowledge Infrastructure (CNKI), Wanfang Data, the VIP Database for Chinese Technical Periodicals (VIP), and the Chinese Biomedical Literature Database (CBM). For Chinese literature, we strictly limited the search to journals indexed in the Peking University Core Directory or Chinese Science Citation Database (CSCD). This restriction was implemented to improve the overall methodological quality of the included studies, as non-core journals in China often lack rigorous reporting of randomization, allocation concealment, and blinding methods, which may introduce a high risk of bias to the meta-analysis. The potential impact of this restriction on publication bias was discussed in the Limitations section.

The search strategy was constructed utilizing a combination of Medical Subject Headings (MeSH) and free-text words, with appropriate adaptations made to accommodate the specific syntax rules of each database. The primary English search terms included: “stroke,” “cerebral infarction,” “cerebral hemorrhage,” “dysphagia,” “deglutition disorders,” “swallowing disorders,” “acupuncture,” “electroacupuncture,” “scalp acupuncture,” “rehabilitation training,” and “randomized controlled trial.” Equivalent Chinese search terms were utilized for the Chinese databases. Furthermore, to prevent the omission of any relevant studies, the reference lists of all eligible full-text articles and previous related reviews were manually cross-checked. Specifically for the Cochrane Library, due to institutional database export limitations at the time, the retrieved records could not be seamlessly imported into the reference management software. Consequently, the initial screening on March 18, 2026, was conducted manually. To provide the detailed itemized screening log, this specific data export was retrospectively reconstructed on August 15, 2026, explicitly utilizing a custom date filter up to the original March 18 cutoff.

### 2.2 Eligibility criteria

#### 2.2.1 Inclusion criteria

①Study Design: Randomized controlled trials (RCTs), regardless of blinding status, published in English or Chinese. ② Participants: Patients meeting the clinical diagnostic criteria for stroke, confirmed by CT or MRI imaging, with concurrent dysphagia. No restrictions were imposed on patient age, sex, disease duration, stroke subtype, or severity. ③ Interventions: The experimental group received acupuncture therapy (either as monotherapy or combined with conventional swallowing rehabilitation training [CSRT]). The control group received matched CSRT (i.e., if the experimental group received acupuncture plus CSRT, the control group received the identical CSRT; if the experimental group received acupuncture alone, the control group received CSRT). Background medical treatments and routine nursing care were kept strictly identical between groups to isolate the specific therapeutic effect of acupuncture. There were no restrictions on acupuncture modalities, acupoint selection, manipulation techniques, treatment frequency, or duration. ④ Outcomes: Studies were required to report at least one of the following core outcomes, either at immediate post-treatment (short-term) or ≥3 months post-treatment (long-term) follow-up: Primary outcomes included the total effective rate of the Water Swallowing Test (WST) (13) and the Standardized Swallowing Assessment (SSA) score (14); Secondary outcomes included the Swallowing Quality of LifeQuestionnaire (SWAL-QOL) score (15) and the incidence of adverse events (AEs). ⑤ No minimum follow-up limit for short-term outcome studies; studies reporting long-term efficacy must have a minimum follow-up duration of 3 months post-treatment.

#### 2.2.2 Exclusion criteria

① Non-RCTs, narrative reviews, case reports, animal studies, mechanistic studies, conference abstracts, and unpublished dissertations. ② Studies in which the control group received acupuncture-related therapies (e.g., acupressure), or the experimental group received additional specific interventions other than acupuncture, precluding the independent extraction of the acupuncture effect. ③ Duplicate publications (only the article with the most comprehensive data and the longest follow-up period was retained). ④ Studies with incomplete data where core outcome metrics could not be obtained even after attempting to contact the corresponding authors. ⑤ Patients presenting with severe cardiac, hepatic, or renal failure, impaired consciousness, severe cognitive impairment, or organic pharyngeal/laryngeal lesions that prevented cooperation with treatment and assessment.

### 2.3 Study selection and data extraction

Records identified through the comprehensive database search were primarily managed using EndNote X21(Clarivate Analytics, Philadelphia, PA, United States). Duplicate records were systematically removed utilizing a combination of EndNote’s automated deduplication tools and rigorous manual cross-checking. Following the removal of duplicate records, the titles and abstracts of the remaining documents were independently screened for initial inclusion. After the initial screening, the fulltexts of the remaining documents were carefully reviewed to evaluate the final inclusion. Two trained reviewers independently screened the literature and extracted data. Discrepancies were resolved through cross-checking and consensus discussions, or by consulting a third reviewer when necessary. A pre-designed, standardized data extraction form was utilized to collect key information, including study characteristics, baseline demographics, intervention protocols, follow-up durations, short- and long-term core outcome data, and elements required for the risk of bias assessment. Corresponding authors were contacted via email to acquire any missing data.

### 2.4 Risk of bias assessment

The methodological quality of the included RCTs was evaluated using the Cochrane Collaboration’s Risk of Bias tool (version 5.1.0). Seven domains were comprehensively assessed: random sequence generation, allocation concealment, blinding of participants and personnel, blinding of outcome assessment, incomplete outcome data, selective reporting, and other biases. Each domain was rigorously graded as having a “low risk,” “unclear risk,” or “high risk” of bias (16).

### 2.5 Statistical analysis

All meta-analyses were performed using Review Manager (RevMan) software, version 5.4. For dichotomous variables (WST total effective rate, AE incidence), the Risk Ratio (RR) was utilized as the effect measure. However, considering the potential for zero-event (single-arm or double-arm) studies regarding extremely rare adverse events, the Risk Difference (RD) was specifically adopted. Continuous variables (SSA and SWAL-QOL scores) were analyzed using the Mean Difference (MD). All effect sizes were reported with their corresponding 95% Confidence Intervals (CIs). Statistical heterogeneity among studies was evaluated using the χ^2^ test and quantified by the *I*^2^ statistic. An *I*^2^ < 50% indicated acceptable heterogeneity, prompting the use of a fixed-effects model; whereas an *I*^2^ ≥ 50% indicated significant heterogeneity, for which a random-effects model was applied, followed by subgroup and sensitivity analyses to explore potential sources of heterogeneity. The core analysis involved a paired comparison between short-term (immediate post-treatment) and long-term (≥3 months post-treatment) outcomes to verify the sustainability of the acupuncture effect. The robustness of the pooled results was evaluated through a “leave-one-out” sensitivity analysis. For outcomes synthesizing data from ≥10 studies, potential publication bias was assessed visually via funnel plots and quantitatively using Egger’s linear regression test, with the significance level set at *α* = 0.05.

### 2.6 Certainty of evidence assessment

The certainty of the evidence for the primary and secondary outcomes was evaluated using the Grading of Recommendations Assessment, Development and Evaluation (GRADE) framework. Two independent reviewers assessed the evidence quality across five domains: risk of bias, inconsistency, indirectness, imprecision, and publication bias. The certainty of evidence for each outcome was ultimately categorized as high, moderate, low, or very low. The GRADEpro Guideline Development Tool (GDT) was utilized to generate the Summary of Findings (SoF) table.

## 3 Results

### 3.1 Literature search and selection

A total of 1,131 potentially relevant records were identified through the initial database searches across eight databases. After the removal of 421 duplicates, 16 clinical trial registry protocols lacking extractable data, and 68 records that failed to meet the language or basic eligibility criteria, 626 articles underwent primary screening based on titles and abstracts. This phase excluded 497 records due to ineligible study designs, target populations, or interventions, leaving 129 articles for full-text review. During the rigorous full-text assessment, 106 articles were further excluded due to confounding interventions, non-RCT designs, lack of long-term follow-up data, or missing core data. Ultimately, 23 RCTs were included in this systematic review, yielding a cumulative sample size of 2,224 patients (1,111 in the experimental group and 1,113 in the control group). The detailed literature screening process is illustrated in Figure 1 (17).

**Figure 1:**
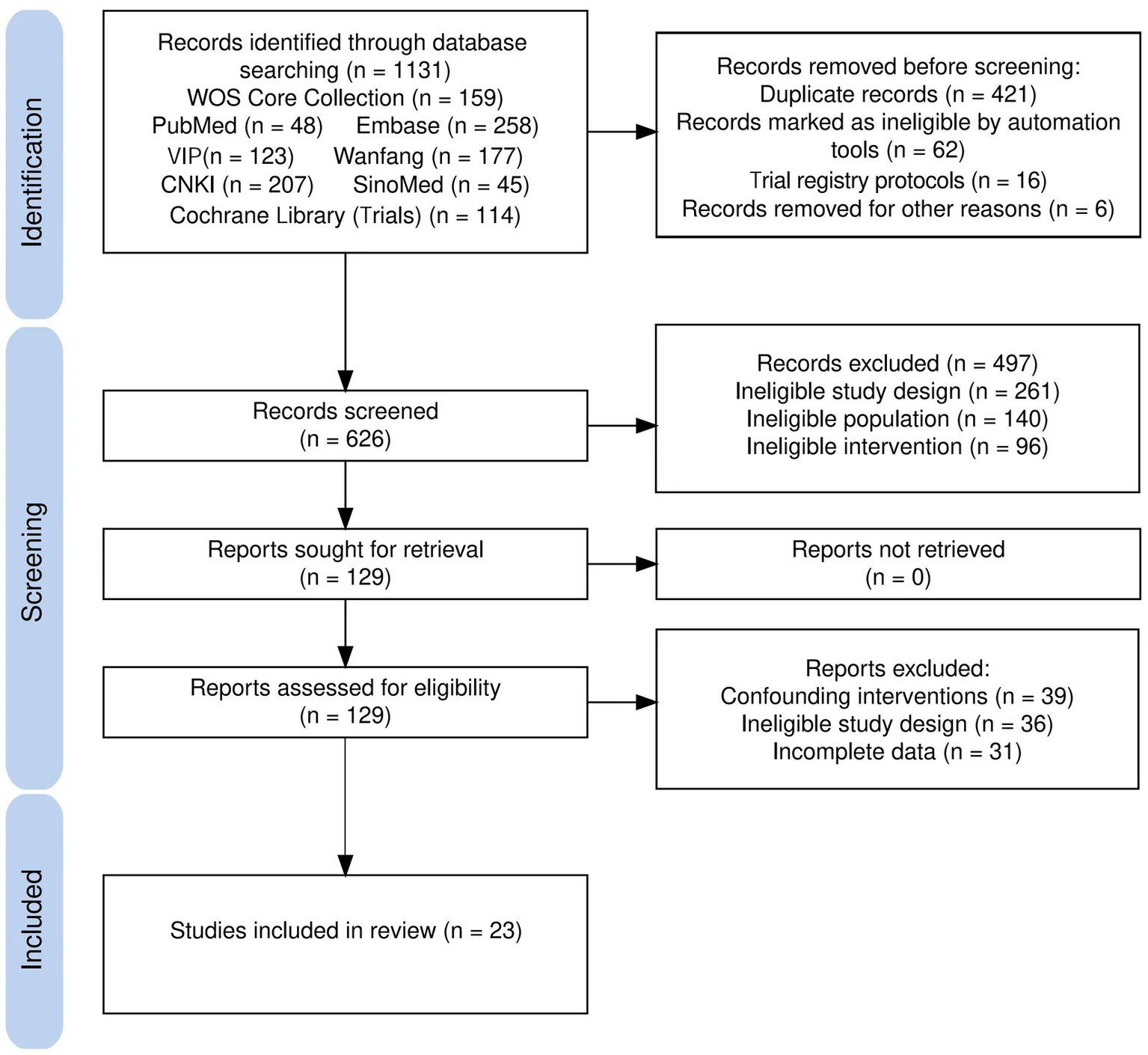
PRISMA flow diagram of the literature search and screening process. CNKI, China National Knowledge Infrastructure; CBM, Chinese Biomedical Literature Database; VIP, VIP Database for Chinese Technical Periodicals; RCT, Randomized Controlled Trial; CSRT, Conventional Swallowing Rehabilitation Therapy.

### 3.2 Study characteristics

The 23 included RCTs consisted of 19 Chinese-language and 4 English-language articles (18–40), collectively encompassing 2,224 patients with confirmed post-stroke dysphagia. Regarding interventions, the experimental groups received acupuncture therapy (either alone or as an adjunct to the control intervention), whereas the control groups primarily received CSRT. Notably, the study by Xia et al. (34) rigorously employed superficial needling at non-acupoints in the control group as a sham acupuncture intervention, providing a robust methodological basis for evaluating the specific therapeutic effects of acupuncture. In terms of follow-up design, 7 studies reported long-term follow-up data at ≥3 months post-treatment. This provided crucial data support for our systematic evaluation of the long-term sustained efficacy of acupuncture in improving post-stroke swallowing function (Table 1)

**Table 1:** Characteristics of included studies.

Study (year)	Sample size (T/C)	Baseline summary	Intervention (T vs. C)	Duration	Primary outcomes
Wang, 2026 (18)	45/45	~68 y; Early phase	T: CMT + Drug + Early Acupuncture C: CMT + Drug	3 weeks	NIHSS, mRS, TCM, WST
Wang, 2026 (19)	102/102	~64 y; Stable phase	T: CSRT + Acupuncture C: CSRT	4 weeks	SSA, WST
Zhang, 2026 (20)	46/46	~69 y; ~12 d duration	T: CMT + CSRT + Acupuncture C: CMT + CSRT	8 weeks	SSA, WST, GUSS, SWAL-QOL
Xu, 2025 (21)	45/45	~61 y	T: CSRT + Body Acupuncture, C: CSRT	4 weeks	SSA, MMASA, WST, SWAL-QOL.
Kang, 2025 (22)	15/15	~63 y; <6 months	T: CSRT + Electroacupuncture C: CSRT	3 weeks	SSA, WST, VFSS, sEMG, FOIS.
Guo, 2024 (23)	40/40	~61 y; 2 w ~ 6 m	T: CSRT + Triple Acupuncture C: CSRT	4 weeks	SSA, WST, sEMG, Mobility.
Wu, 2024 (24)	65/62	~66 y; 15 d ~ 6 m	T: CSRT + Posture control + EA C: CSRT	4 weeks	SSA, WST, Complication rate
Cao, 2023 (25)	30/30	~60 y; 14 ~ 62 d	T: NMES + Four-step Acupuncture C: NMES	4 weeks	SSA, WST, PAS
Cheng, 2022 (26)	18/20	~62 y; ~55 d	T: CMT + Penetrating/Filiform Acu. C: CMT + CSRT	4 weeks	SSA, SpO2, Albumin, Prealbumin
Bai, 2022 (27)	30/30	~65 y; 1 ~ 3 months	T: CSRT + Tongdu Acu. C: CSRT	2 weeks	SSA, SpO2, Hb, Serum ALB
Gao, 2020 (28)	30/30	~64 y; 0.2 ~ 6 months	T: CMT + CSRT + Nape Cluster Acu. C: CMT + CSRT	4 weeks	SSA, VFSS, sEMG, US params
Wang, 2020 (29)	74/73	~63 y; 14 ~ 65 d	T: CMT + CSRT + EA C: CMT + CSRT	4 weeks	WST, SWAL-QOL
Qin, 2019 (30)	52/52	~53 y; 3 ~ 9 months	T: CSRT + Deep EA C: CSRT	4 weeks	SSA, WST
Li, 2019 (31)	40/40	~62 y	T: CMT + CSRT + Nape Acu. C: CMT + CSRT	4 weeks	SSA, WST, PAS, FEES
Chu, 2017 (32)	48/49	~67 y; <6 months	T: CMT + CSRT + Nape Acu. C: CMT + CSRT	8 weeks	SSA, SWAL-QOL, RSST
Xia, 2016 (33)	60/60	~65 y; ~9 d	T: CSRT + Combined Acu. C: CSRT	4 weeks	SSA, DOSS, MBI, SWAL-QOL
Xia, 2016 (34)	61/55	~66 y	T: CMT + CSRT + Syndrome Acu. C: CMT + CSRT + Sham Acu.	6 weeks	SSA, VFSS, MBI, SWAL-QOL
Yang, 2011 (35)	35/35	~67 y; Acute phase	T: CMT + CSRT + EA C: CMT + CSRT	3 weeks	SSA
Xie, 2011 (36)	70/70	~66 y; ≤30 d	T: CMT + Alternating Acu./EA C: CMT + CSRT	2–4 weeks	WST, Kubota, Infection rate.
Wu, 2011 (37)	75/75	~67 y	T: CSRT + EA C: CSRT	4 weeks	TCM, WST, SWAL-QOL
Xie, 2011 (38)	70/70	~66 y; 30 ~ 180 d	T: CSRT + Body/Scalp EA C: CSRT	4 weeks	WST, Kubota
Huang, 2010 (39)	32/30	~54 y; 20 ~ 42 d	T: CMT + CSRT + EA C: CMT + CSRT	4 weeks	WST, Expectoration, VFSS
Zhou, 2006 (40)	34/33	47–79 y	T: CMT + EA C: CMT	15 days	WST, Cure time

Group: T, Treatment group; C, Control group; y, years; d, days.

Interventions: CMT, Conventional medical treatment; CSRT, Conventional swallowing rehabilitation therapy; NMES, Neuromuscular electrical stimulation; EA, Electroacupuncture; Acu., Acupuncture.

Outcomes: WST, Water Swallowing Test; SSA, Standardized Swallowing Assessment; SWAL-QOL, Swallowing Quality of Life; VFSS, Videofluoroscopic Swallowing Study; sEMG, Surface electromyography; PAS, Penetration-Aspiration Scale.

### 3.3 Risk of bias in included studies

Among the 23 included RCTs, 17 studies explicitly reported correct methods for random sequence generation and were assessed as having a low risk of bias, while the remaining 6 studies did not detail their randomization methods and were rated as unclear risk. Only 5 studies clearly described the implementation of allocation concealment (low risk), whereas the other 18 studies lacked relevant descriptions (unclear risk). Only 1 study successfully implemented double-blinding for participants, personnel, and outcome assessors (low risk); the remaining 22 studies were unable to double-blind participants and operators due to the operational characteristics of acupuncture interventions, and were thus rated as having a high risk of performance bias. Regarding the blinding of outcome assessors, 9 studies explicitly implemented it (low risk), while the remaining 14 studies did not describe it (unclear risk). All studies maintained complete outcome data, yielding a low risk of attrition bias. Furthermore, all studies fully reported their prespecified outcome measures without evidence of selective reporting (low risk). Seven studies did not explicitly report detailed statistical results for baseline comparability (unclear risk), while the remaining 16 studies demonstrated well-balanced baselines (Figures 2, 3).

**Figure 2:**
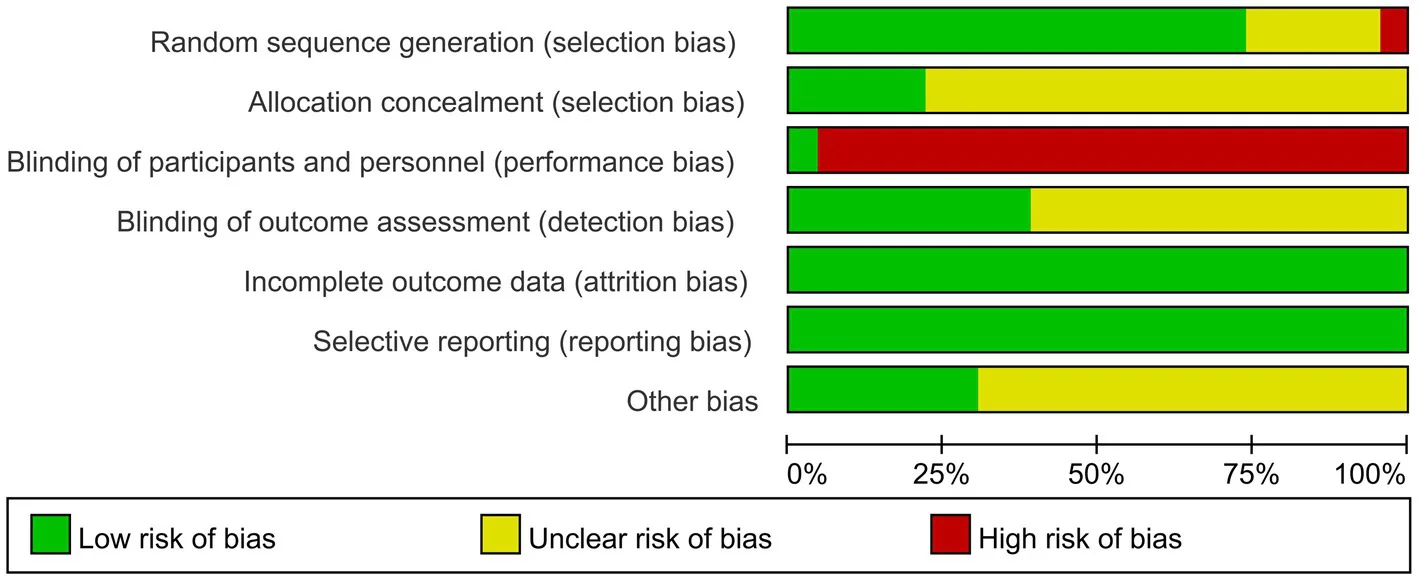
Risk of bias summary of included studies.

**Figure 3:**
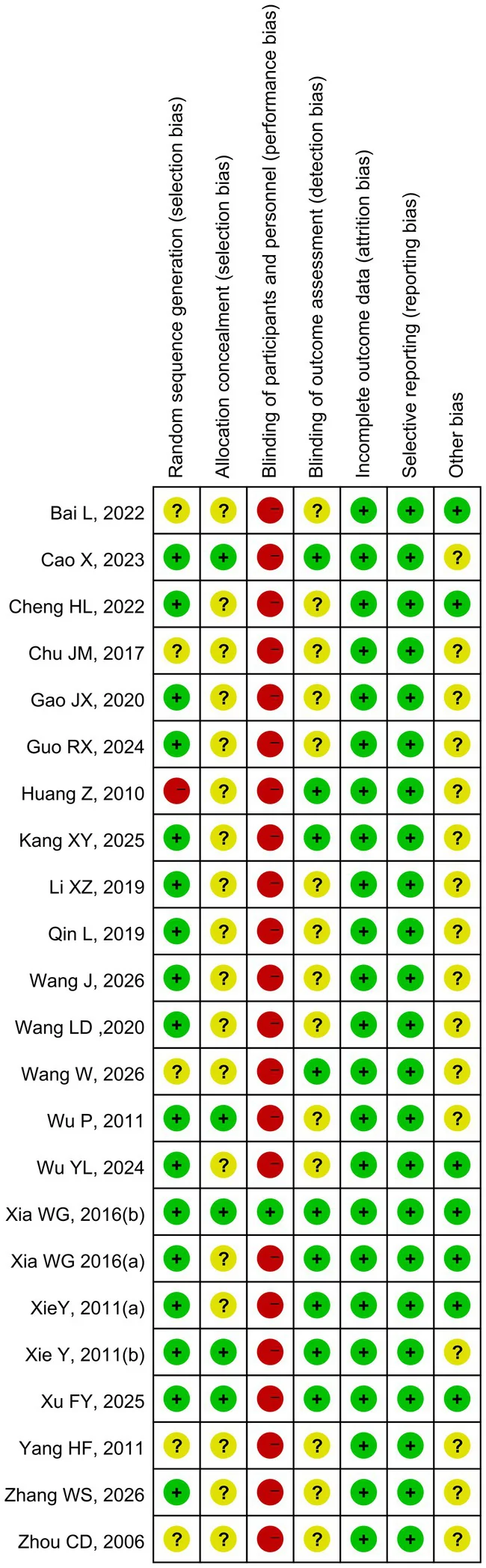
Risk of bias details for each included study (+: low risk?: unclear risk; −: high risk).

### 3.4 Meta-analysis results (core comparison of short- and long-term efficacy)

#### 3.4.1 Primary outcomes

##### 3.4.1.1 Water swallowing test (WST)

A rigorous data homogenization process was applied to the clinical effective rate of the WST. The WST scale comprises Grades I to V. Given the discrepancies in the definition of “effective” across the original studies, we standardized the efficacy criteria during data extraction to eliminate bias derived from this heterogeneity: only patients categorized as Grade I (drinking in one gulp without choking) and Grade II (drinking in two or more gulps without choking) were uniformly defined as “effective”; conversely, patients categorized as Grade III, IV, and V (accompanied by varying degrees of choking or inability to swallow) were uniformly defined as “ineffective.” The events of efficacy/inefficacy in all original studies were recalculated and extracted based on this unified standard.

Short-term efficacy: A total of 13 studies were included. The pooled results using a random-effects model demonstrated that the short-term WST effective rate in the acupuncture combined with CSRT group was significantly higher than that in the CSRT-alone group (RR = 1.50, 95% CI [1.31, 1.73], *p <* 0.001, *I*^2^ = 65%) (Figure 4).

**Figure 4:**
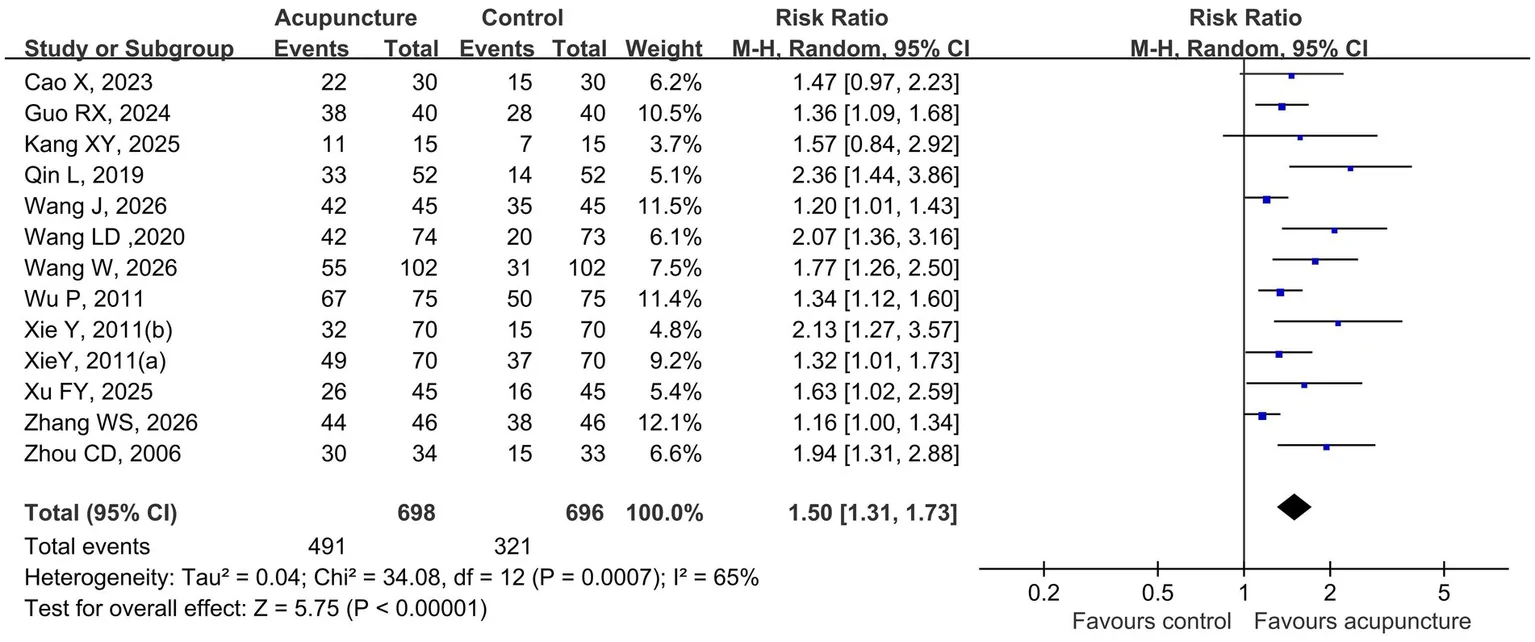
Forest plot of the short-term WST total effective rate for acupuncture in treating post-stroke dysphagia.

Long-term efficacy: Five studies reported the WST effective rate during the follow-up period (≥ 3 months). Data synthesis confirmed that several months after treatment cessation, the acupuncture group still exhibited a significant long-term advantage in maintaining efficacy (RR = 1.38, 95% CI [1.21, 1.58], *p <* 0.001), with low inter-study heterogeneity (*I*^2^ = 35%) (Figure 5).

**Figure 5:**
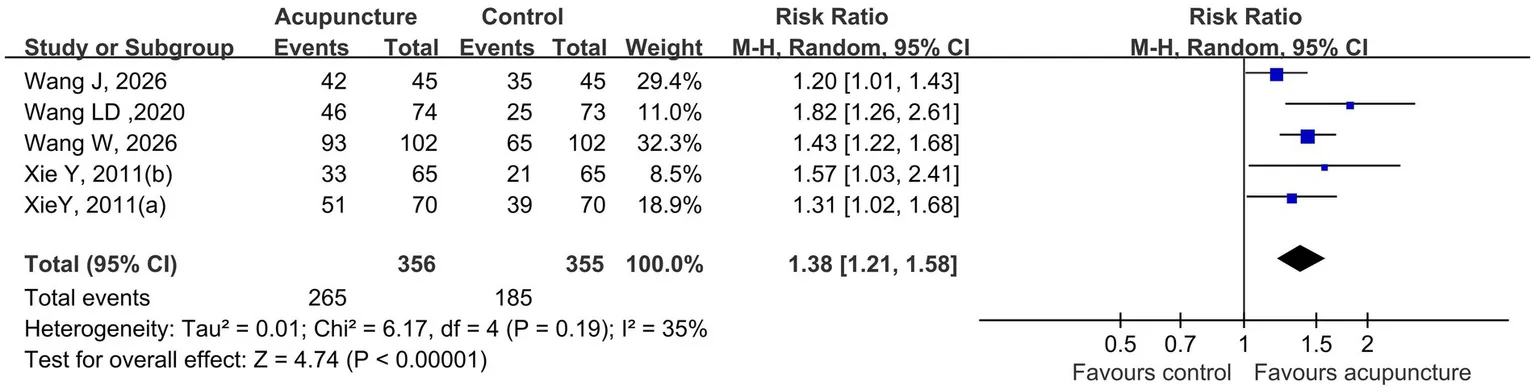
Forest plot of the long-term WST total effective rate.

Comparative analysis of short- and long-term efficacy: Comparing the effect sizes at different time points reveals that the high clinical effective rate achieved immediately after the intervention (RR = 1.50) was excellently maintained ≥3 months after treatment cessation (RR = 1.38). Although the long-term relative risk exhibited an extremely marginal numerical decline, the 95% confidence intervals of the two endpoints highly overlapped, and the statistical significance of the long-term efficacy remained extremely robust (*p <* 0.001). However, it must be acknowledged that the five studies contributing to the long-term WST pool represent a strict subset of the short-term study cohort rather than entirely independent replications. Nonetheless, this trajectory strongly indicates that the awakening effect of acupuncture on the swallowing reflex and airway protection mechanisms is not a transient response, but rather possesses a definitive and stable long-term sustained effect within these followed-up patients.

##### 3.4.1.2 Standardized swallowing assessment (SSA) score

Short-term efficacy: Eleven studies, encompassing 925 patients (464 in the experimental group and 461 in the control group), reported SSA scores immediately post-treatment. Meta-analysis using a random-effects model indicated that acupuncture significantly reduced short-term SSA scores, demonstrating a superior improvement in swallowing function compared to the control group (MD = −3.33, 95% CI [−4.46, −2.19], *p* < 0.001). However, substantial statistical heterogeneity was observed among the included studies (*I*^2^ = 82%) (Figure 6).

**Figure 6:**
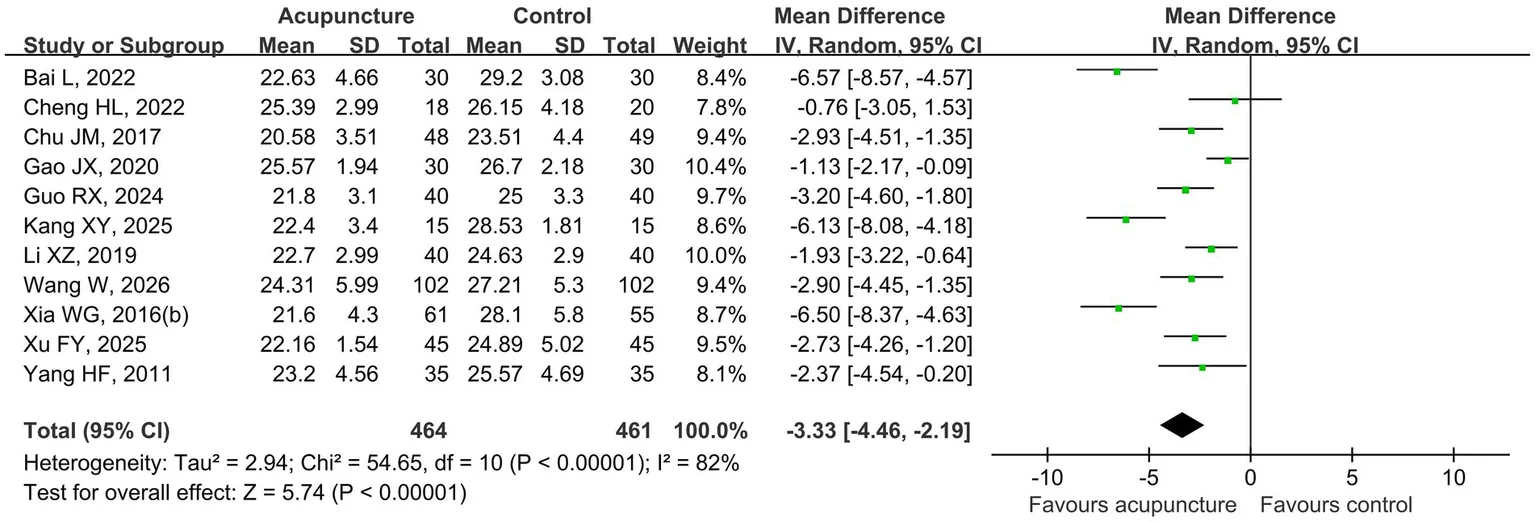
Forest plot of short-term SSA scores.

Long-term efficacy: Two studies provided long-term follow-up SSA scores. Pooled results using a fixed-effects model revealed that at ≥3 months post-treatment, the SSA score in the acupuncture group remained persistently and significantly lower than that in the control group (MD = −4.92, 95% CI [−6.54, −3.31], *p* < 0.001). Notably, these studies exhibited absolute statistical homogeneity (*I*^2^ = 0%), providing highly robust evidence for the long-term stability of acupuncture in improving swallowing function (Figure 7).

**Figure 7:**
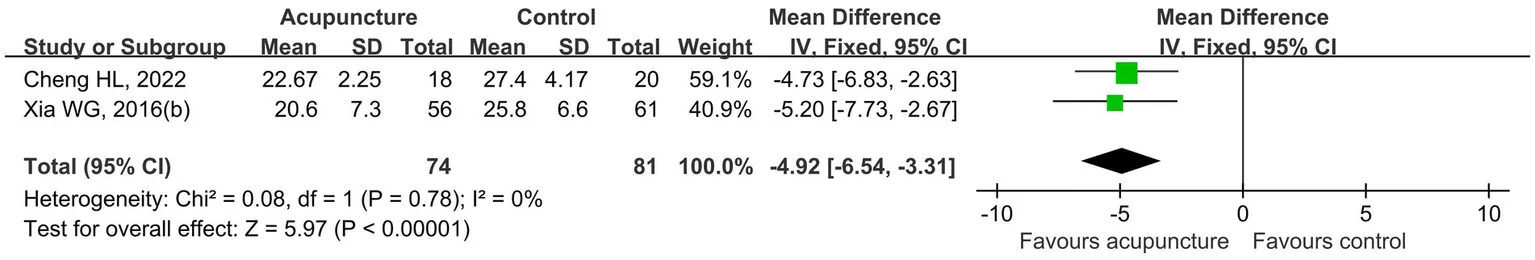
Forest plot of long-term SSA scores.

Comparative analysis of short- and long-term efficacy: Comparing the effect trajectories at different time points yields important clinical insights. However, it must be explicitly noted that the two studies providing long-term follow-up SSA scores are a strict subset of the short-term pool, rather than independent replications. While the magnitude of the pooled SSA score reduction at ≥3 months post-treatment (MD = −4.92) was numerically superior to the overall pooled effect size of the short-term meta-analysis (MD = −3.33), this represents a sustained effect within this specific cohort rather than a broad cross-validation. Nevertheless, these findings suggest that the functional recovery induced by acupuncture may be maintained over time within these populations, though larger independent long-term trials are required.

#### 3.4.2 Secondary outcomes

##### 3.4.2.1 SWAL-QOL score

Short-term efficacy: Six studies evaluated patients’ short-term SWAL-QOL scores. The results showed that the acupuncture group significantly improved SWAL-QOL scores compared to the control group (MD = 21.58, 95% CI [14.79, 28.37], *p <* 0.001, *I*^2^ = 79%) (Figure 8).

**Figure 8:**
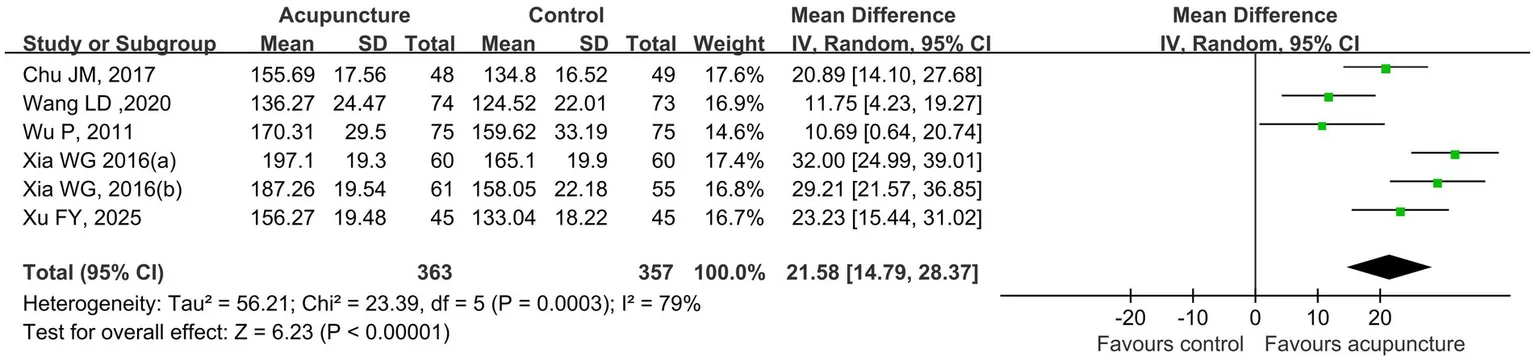
Forest plot of short-term SWAL-QOL scores.

Long-term efficacy: Two studies provided long-term quality of life follow-up data. Pooled results using a random-effects model showed that the degree of long-term improvement in the acupuncture group remained significantly superior to the control group (MD = 23.56, 95% CI [5.66, 41.46], *p =* 0.010). Although there was considerable statistical heterogeneity for this indicator (*I*^2^ = 88%, Figure 9), the direction of effect in both independent studies was highly consistent (both favoring the acupuncture group). Due to the limited number of included studies (*k* = 2), sensitivity analyses and funnel plot evaluations were not performed for this indicator below.

**Figure 9:**
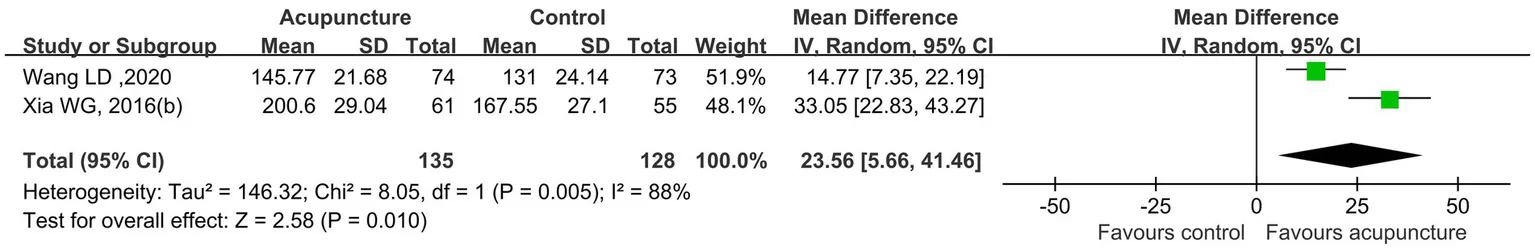
Forest plot of long-term SWAL-QOL scores.

Comparative analysis of short- and long-term efficacy: The improvement in patients’ subjective quality of life demonstrated a time trajectory consistent with objective swallowing functional assessments. The short-term pooled effect size (MD = 21.58) and the long-term pooled effect size (MD = 23.56) were numerically similar. As with the SSA outcomes, the long-term SWAL-QOL data is derived from a two-study subset of the short-term pool. Thus, while the data suggests that enhancements in quality of life—encompassing fear of eating, mental health, and social adaptation—can be maintained in these cohorts after treatment cessation, the durability of these effects should be interpreted cautiously pending further large-scale validation.

##### 3.4.2.2 Safety analysis

Eight studies explicitly reported acupuncture-related adverse events (AEs). The majority of reported AEs (such as localized subcutaneous hematoma, transient needling pain, or fainting) were specific to acupuncture manipulation. With the exception of one study [Xia et al. (34)] that utilized superficial non-acupoint needling as a sham control, the control groups in the remaining studies did not receive any sham intervention, presenting typical “single-arm zero-event” data characteristics. Therefore, a fixed-effects model (*I*^2^ = 41%) was employed to quantitatively synthesize the Risk Difference (RD). Meta-analysis revealed that the incidence of AEs in the combined acupuncture group was higher than in the CSRT-alone group, with an absolute risk difference of 11% (RD = 0.11, 95% CI [0.08, 0.14], *p <* 0.001). However, the original data indicated that all aforementioned adverse reactions were extremely mild local physical stress responses that resolved spontaneously without requiring special medical intervention, and no severe adverse events (SAEs) occurred. Coupled with its significant improvement in swallowing function, acupuncture exhibits a highly favorable overall clinical safety profile for treating PSD (Figure 10).

**Figure 10:**
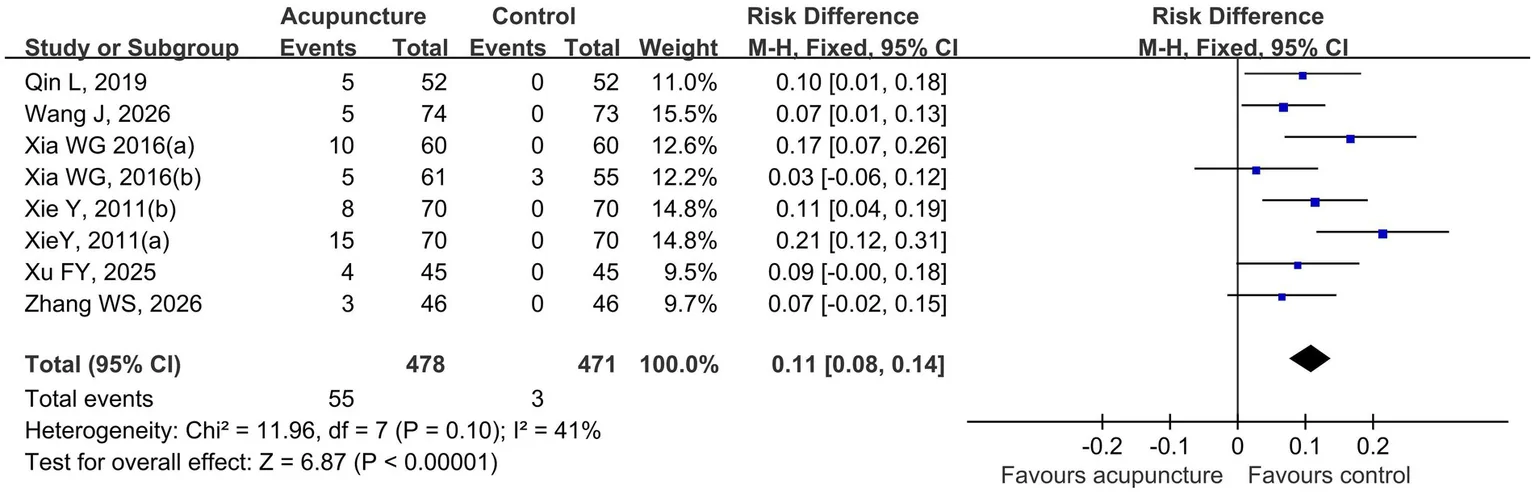
Forest plot of the incidence of adverse reactions.

### 3.5 Subgroup analysis

Based on the prespecified protocol, subgroup analyses were conducted for the short-term WST effective rate and short-term SSA scores—which exhibited higher heterogeneity—using “intervention modality (electroacupuncture vs. manual acupuncture)” and “stroke phase (acute vs. recovery phase)” as covariates.

Regarding the short-term WST effective rate, subgroup analysis revealed that the effect size of the electroacupuncture group (RR = 1.68, 95% CI [1.37, 2.07]) was higher than that of the manual acupuncture group (RR = 1.34, 95% CI [1.15, 1.56]). The test for subgroup differences indicated borderline statistical significance (*p =* 0.08), suggesting a marginal potential advantage for electroacupuncture, though it does not establish definitive superiority. The intra-subgroup heterogeneity for both groups (*I*^2^ = 53 and 56%) decreased to varying degrees compared to the overall heterogeneity (*I*^2^ = 65%) (Figure 11).

**Figure 11:**
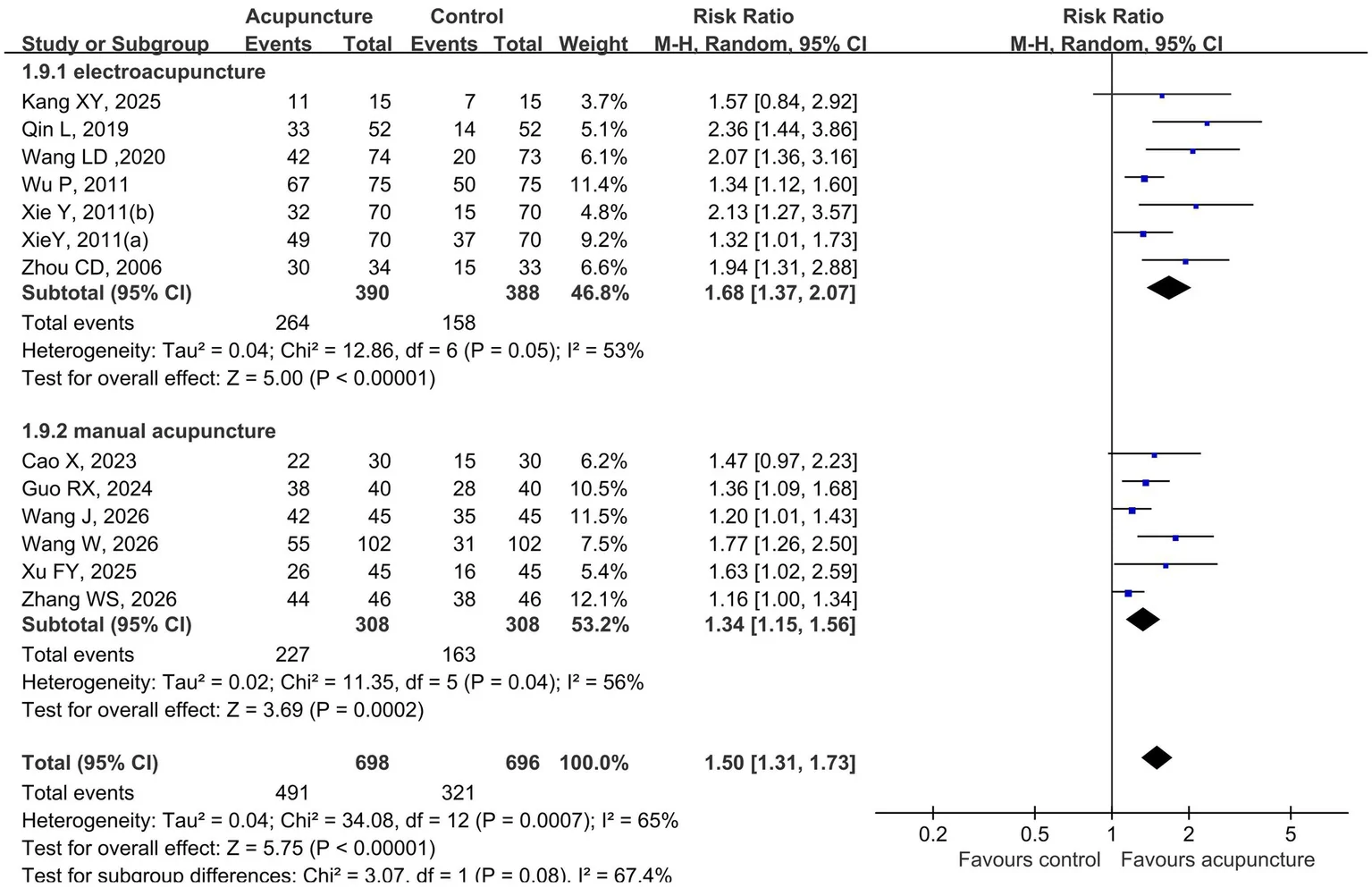
Forest plot of subgroup analysis for short-term WST based on the use of electroacupuncture.

For short-term SSA scores, focusing on immediate therapeutic effects ensured an adequate study count for robust comparison between stimulation modalities. Both electroacupuncture and manual acupuncture groups significantly reduced short-term SSA scores. After incorporating the latest studies into the manual acupuncture subgroup, the test for subgroup differences yielded a *p*-value of 0.56 (*p* > 0.1). This confirms that no statistically significant superiority exists between the two modalities for this objective functional outcome, and suggests that the stimulation modality is not the primary source of heterogeneity for the SSA outcome (Figure 12).

**Figure 12:**
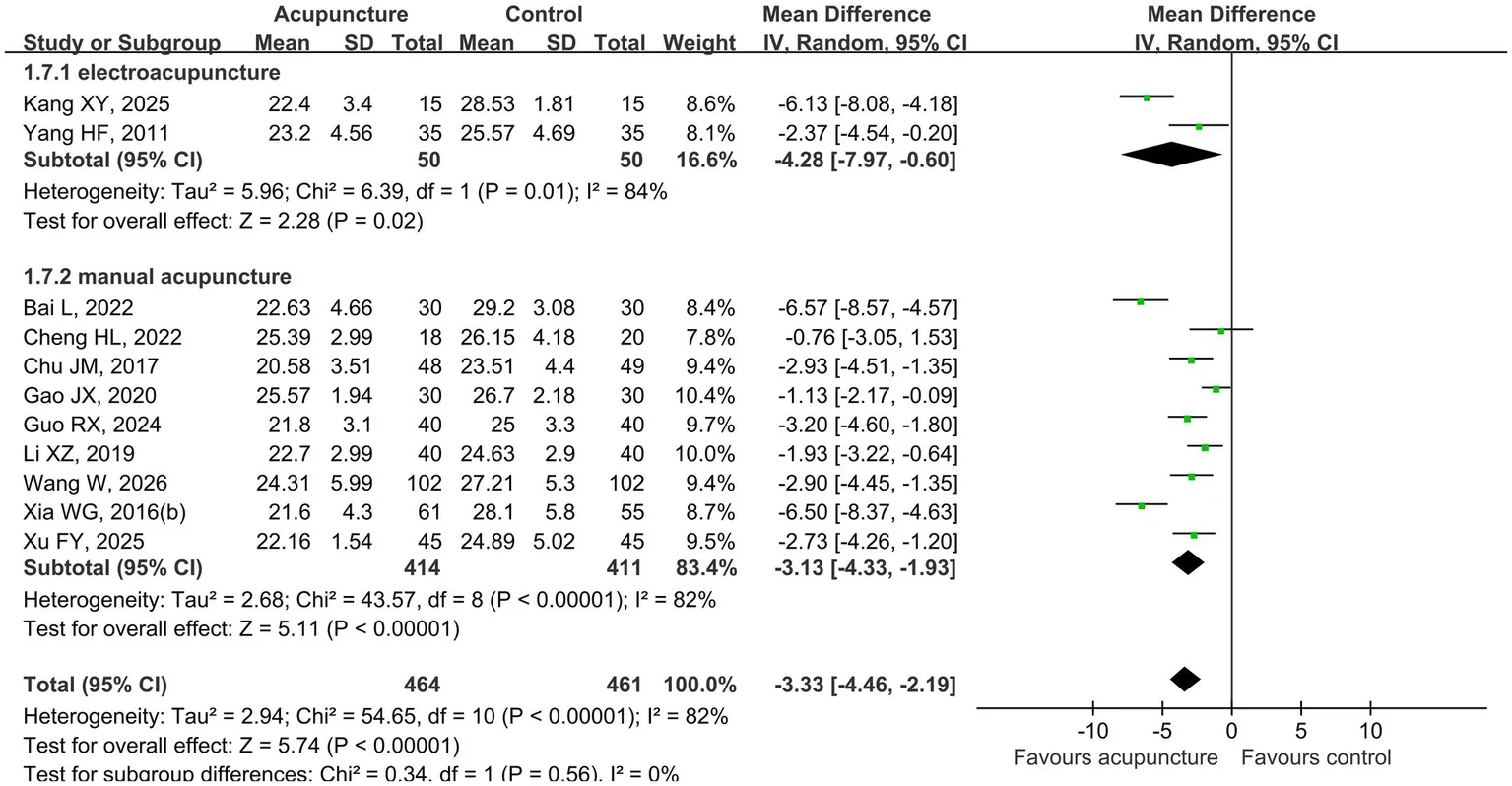
Forest plot of subgroup analysis for short-term SSA based on the use of electroacupuncture.

In exploring the timing of intervention, this study utilized 14 days (2 weeks) as the core clinical cutoff point for scientific stratification. This precisely aligns with the operational time window defined by the American Heart Association/American Stroke Association (AHA/ASA) Guidelines for the Early Management of Patients With Acute Ischemic Stroke (41), which designates the first 2 weeks post-stroke as the early acute management phase: studies strictly limiting inclusion to ≤14 days were classified as the “Acute phase” group;studies where patients had reached a stable phase or the intervention window explicitly fell between 14 days and 6 months were classified as the “Recovery phase” group. Mixed-duration studies with excessively large spans were grouped into an independent subgroup to prevent data contamination. However, regarding the timing of intervention (stroke phase), a subgroup analysis could only be feasibly conducted for the short-term WST. For the SSA scores, the vast majority of the included RCTs (6 out of 9) utilized broad inclusion criteria for disease duration (e.g., “<3 months” or “within 6 months”), leading to heavily mixed patient populations. Furthermore, only a single trial exclusively enrolled patients strictly in the acute phase (≤14 days). This inadequate number of studies per stratum statistically precludes the execution of a valid subgroup meta-analysis and the evaluation of inter-subgroup differences.

Results showed that the pooled relative risk for the short-term WST in the acute phase group (disease duration ≤14 days) was 1.18 (95% CI [1.05, 1.31], *p =* 0.005); whereas the pooled effect size for the subacute and recovery phase group (disease duration 14 days to 6 months) surged to 1.57 (95% CI [1.39, 1.79], *p <* 0.001). The test for subgroup differences showed high statistical significance (*p =* 0.002). Crucially, after stratifying by this clinical feature, the statistical heterogeneity (*I*^2^) within the acute phase and recovery phase subgroups dropped precipitously to 0 and 11%, respectively. This confirms with high certainty that the stroke phase during which acupuncture is initiated is the core source of clinical heterogeneity for the short-term WST clinical efficacy (Figure 13).

**Figure 13:**
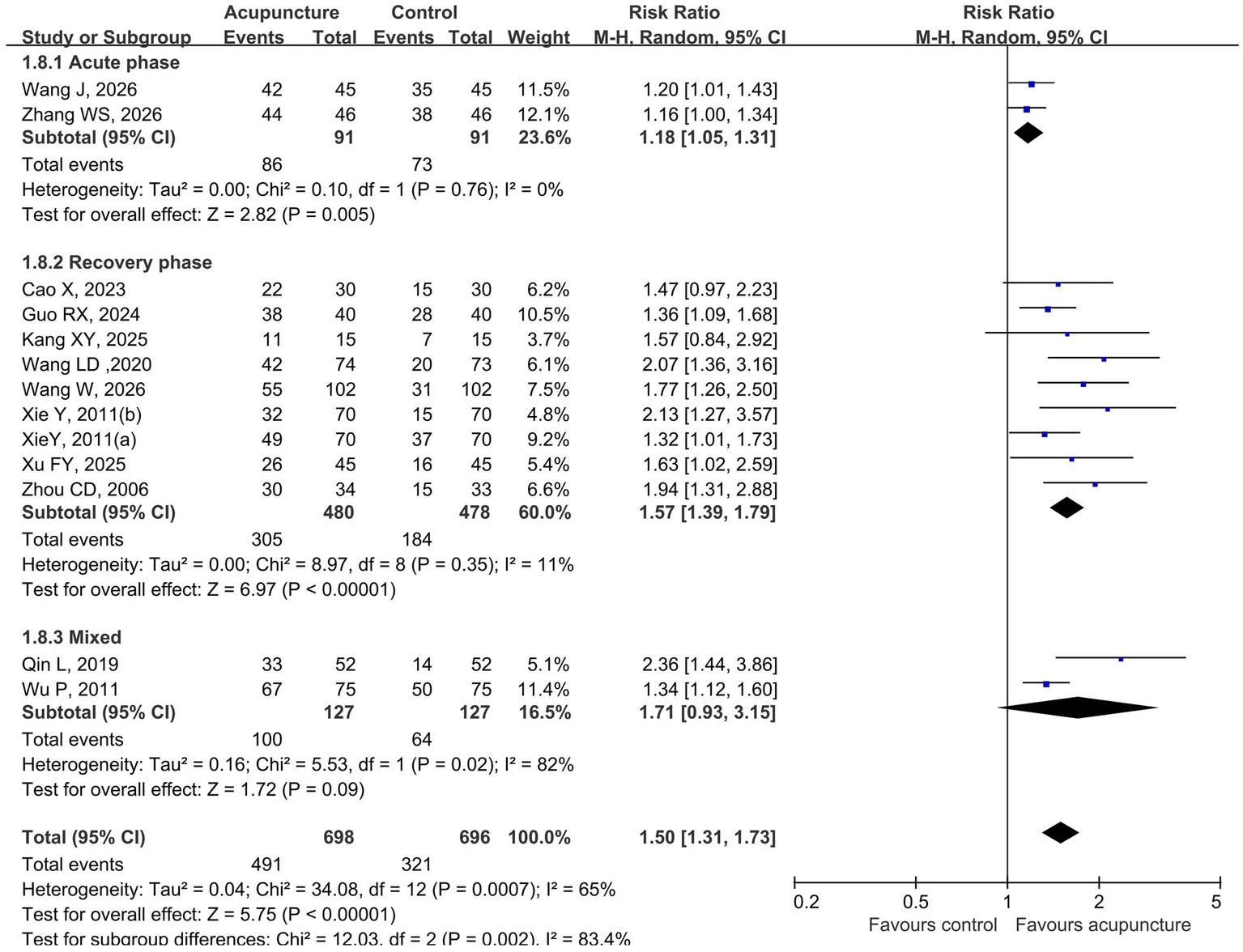
Forest plot of subgroup analysis for short-term WST based on different acupuncture timing.

### 3.6 Sensitivity analysis

For the short-term SSA score (*I*^2^ = 82%) and short-term SWAL-QOL score (*I*^2^ = 79%), which exhibited substantial statistical heterogeneity, a rigorous sensitivity analysis was performed. The results demonstrated that after sequentially omitting any single study, the direction of the pooled effect sizes for both indicators remained consistently unchanged, and the differences consistently maintained robust statistical significance (*p* < 0.05), indicating that our core conclusions regarding the efficacy of acupuncture are highly stable.

Regarding the specific sources of heterogeneity for the short-term SSA score, further sensitivity testing revealed that upon simultaneously excluding the studies by Bai et al. (27), Xia et al. (34), and Kang et al. (22), the statistical heterogeneity decreased precipitously (*I*^2^ = 28%). This strongly suggests that these three specific trials were the primary drivers of the observed heterogeneity, which may be clinically attributable to their distinct variations in acupoint selection, high-frequency electroacupuncture parameters, or specific baseline severity of the enrolled patients.

Furthermore, to address the potential clinical heterogeneity arising from different intervention designs, we conducted a specific sensitivity analysis by excluding the only two trials that utilized acupuncture as a head-to-head monotherapy without conventional rehabilitation [Cheng et al. (26) for SSA; Xie et al. (36) for WST]. Notably, the exclusion of these non-add-on trials did not resolve the statistical heterogeneity, as the *I*^2^ value shifted slightly from 65 to 69% for short-term WST and remained constant at 82% for short-term SSA. However, this exclusion did not materially alter the pooled effect sizes or the statistical significance of the remaining add-on therapy pool. This confirms that while statistical heterogeneity persists across the included studies, our primary efficacy findings are highly robust and are not driven by the inclusion of these distinct clinical designs.

### 3.7 Publication bias

For the “short-term WST effective rate” outcome measure, which encompasses 13 studies (≥10 included articles), this study generated a funnel plot and applied Egger’s linear regression test to evaluate potential publication bias. Visual inspection revealed that the effect points of the individual studies exhibited obvious asymmetry in the funnel plot, with a distinct absence of scatter points in the lower left region (indicating a lack of small-sample studies with smaller effect sizes or negative results, Figure 14). Further quantitative testing confirmed this visual observation (Egger’s test, *p* < 0.001), demonstrating the presence of statistically significant publication bias (small-study effects) for this specific outcome.

**Figure 14:**
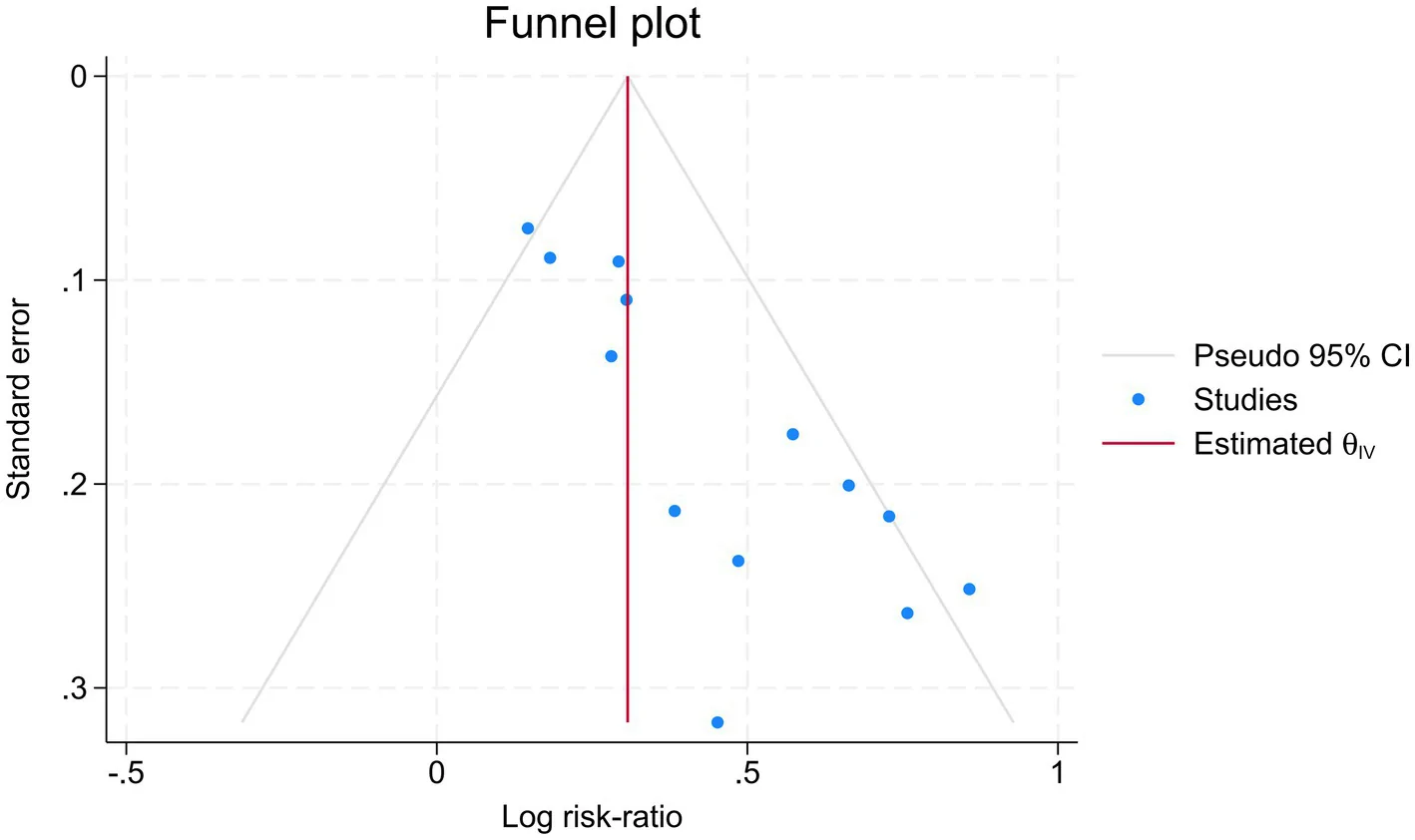
Funnel plot for the evaluation of publication bias underlying the short-term WST effective rate.

Conversely, for the “short-term SSA score” outcome, which now successfully includes 11 studies, an assessment for publication bias was also performed. Visual inspection of the funnel plot revealed a relatively symmetrical distribution of the included studies around the pooled effect estimate, without obvious clustering or missing areas of scatter points (Figure 15). This visual observation was further corroborated by the quantitative analysis using Egger’s regression test, which yielded a *p*-value of 0.250 (*p* > 0.05), indicating no evidence of significant publication bias. This crucial finding suggests that the pooled results for the objective, continuous SSA metric are statistically robust and significantly less susceptible to the small-study effects observed in the dichotomous WST data.

**Figure 15:**
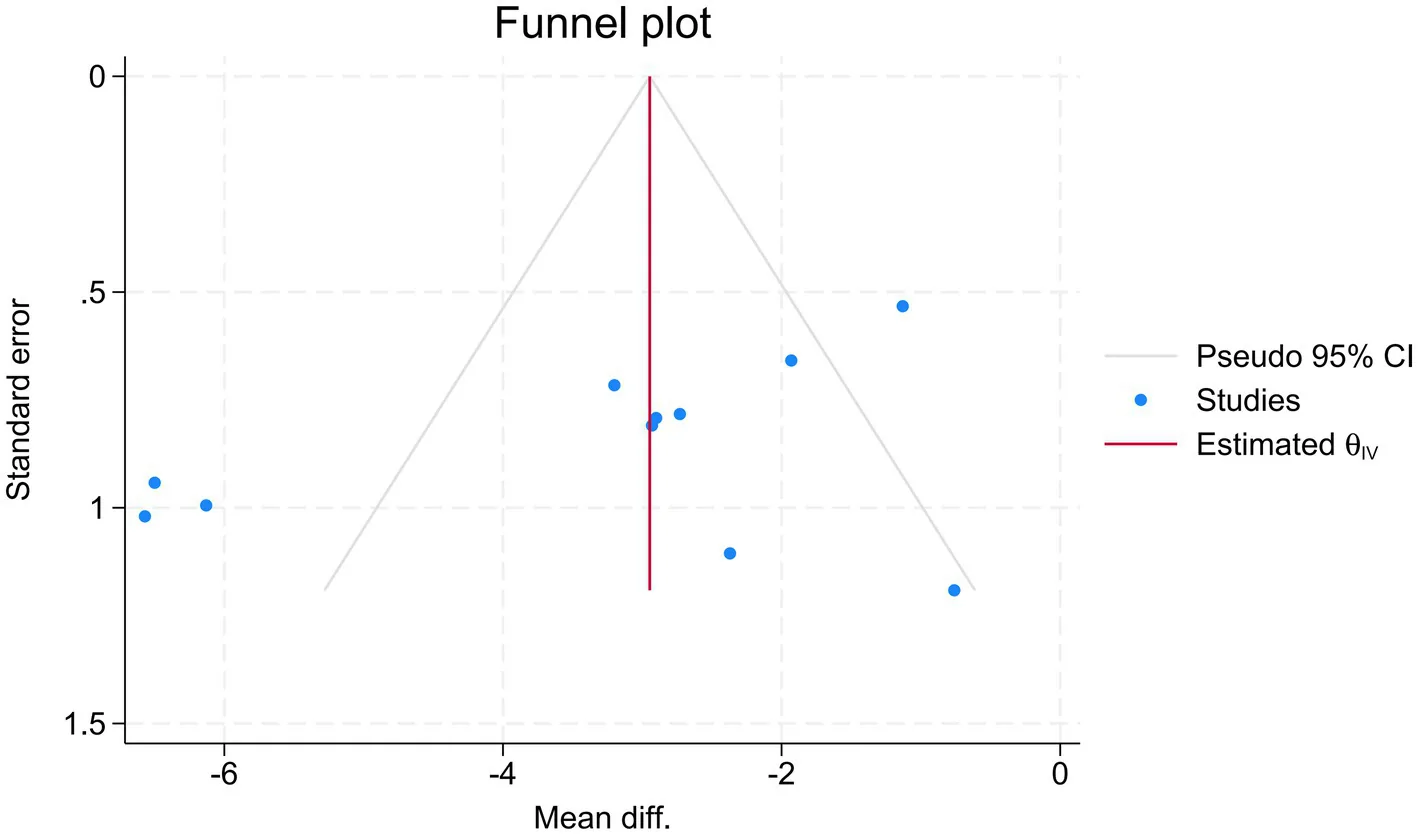
Funnel plot for the evaluation of publication bias underlying the short-term SSA.

### 3.8 Certainty of evidence

The certainty of evidence across all evaluated outcomes was comprehensively assessed using the GRADE framework. Following rigorous re-evaluation, the incidence of adverse events, short-term SSA scores, and short-term SWAL-QOL scores were rated as moderate certainty. Although statistical heterogeneity was observed in the latter two outcomes, all point estimates consistently favored the acupuncture group without contradictory clinical directions; therefore, evidence was not downgraded for inconsistency. These outcomes were primarily downgraded by one level solely due to the inherent high risk of performance bias, as strict double-blinding is not feasible in clinical acupuncture trials.

Several outcomes were evaluated as low certainty. Specifically, the certainty for the long-term WST effective rate and long-term SSA score was rated as low; in addition to the inherent performance bias, they were further downgraded by one level for imprecision due to the limited number of follow-up studies and restricted cumulative sample sizes, which failed to meet the optimal information size threshold. Similarly, the short-term WST effective rate was rated as low certainty due to high risk of bias and confirmed publication bias (Egger’s test, *p* < 0.001).

Furthermore, the certainty for the long-term SWAL-QOL score was assessed as very low, driven by a combination of high risk of bias and very serious imprecision stemming from the extremely small sample size in the long-term subjective assessment pool. A detailed summary of the findings and the specific rationale for each downgrade are comprehensively presented in Table 2.

**Table 2:** Summary of findings (SoF): efficacy and safety of acupuncture for post-stroke dysphagia.

Certainty assessment								Summary of findings					Importance
Outcomes	№ of studies	Study design	Risk of bias	Inconsistency	Indirectness	Imprecision	Other considerations	Patients (Acupuncture)	Patients (Control)	Relative effect (95% CI)	Absolute effect (95% CI)	Certainty	
Short-term WST effective rate	13	Randomized trials	Seriousᵃ	Not serious	Not serious	Not serious	Publication biasᵇ	491/698 (70.3%)	321/696 (46.1%)	RR 1.50 (1.31 to 1.73)	231 more per 1,000 (from 143 more to 337 more)	⨁⨁◯◯ LOWᵃ^,^ᵇ	CRITICAL
Long-term WST effective rate (≥3 months)	5	Randomized trials	Seriousᵃ	Not serious	Not serious	Serious ᶜ	None	265/356 (74.4%)	185/355 (52.1%)	RR 1.38 (1.21 to 1.58)	198 more per 1,000 (from 109 more to 302 more)	⨁⨁◯◯ LOWᵃ^,^ᶜ	CRITICAL
Short-term SSA score	11	Randomized trials	Seriousᵃ	Not serious	Not serious	Not serious	None	464	461	-	MD 3.33 lower (4.46 lower to 2.19 lower)	⨁⨁⨁◯ MODERATEᵃ	CRITICAL
Long-term SSA score (≥3 months)	2	Randomized trials	Seriousᵃ	Not serious	Not serious	Serious ᶜ	None	74	81	-	MD 4.92 lower (6.54 lower to 3.31 lower)	⨁⨁◯◯ LOWᵃ^,^ᶜ	CRITICAL
Short-term SWAL-QOL score	6	Randomized trials	Seriousᵃ	Not serious	Not serious	Not serious	None	363	357	-	MD 21.58 higher (14.79 higher to 28.37 higher)	⨁⨁⨁◯ MODERATEᵃ	IMPORTANT
Long-term SWAL-QOL score (≥3 months)	2	Randomized trials	Seriousᵃ	Not serious	Not serious	Very serious ᵈ	None	135	128	-	MD 23.56 higher (5.66 higher to 41.46 higher)	⨁◯◯◯ VERY LOWᵃ^,^ᵈ	IMPORTANT
Adverse events	8	Randomized trials	Seriousᵃ	Not serious	Not serious	Not serious	None	55/478 (11.5%)	3/471 (0.6%)	Not estimable	110 more per 1,000 (from 80 more to 140 more)	⨁⨁⨁◯ MODERATEᵃ	IMPORTANT

CI, Confidence interval; RR, Risk ratio; MD, Mean difference; RCTs, Randomised controlled trials.

^a^Downgraded one level for risk of bias: High risk of performance bias due to the inherent impossibility of double-blinding in acupuncture interventions; unclear allocation concealment in most studies.

^b^Downgraded one level for publication bias: Asymmetrical funnel plot and Egger’s test indicated highly suspected publication bias (*p* < 0.001).

^c^Downgraded one level for imprecision: The total sample size did not meet the Optimal Information Size (OIS) threshold (fewer than 400), leading to wide confidence intervals that may cross the threshold of clinical decision-making.

^d^Downgraded two levels for imprecision: Very small total sample size and excessively wide confidence intervals crossing the absolute effect thresholds for meaningful clinical benefit.

⊕○○○ Very Low: The true effect is probably markedly different from the estimated effect with very little confidence in the effect estimate; ⊕ ⊕ ○○ Low: The true effect is likely to be close to the estimated effect, but there is a possibility that it is substantially different; ⊕ ⊕ ⊕○ Moderate: The true effect is likely to be close to the estimated effect, with moderate confidence in the effect estimate.

## 4 Discussion

### 4.1 Summary of principal findings

This comprehensive systematic review and meta-analysis, encompassing 23 RCTs and 2,224 patients, systematically quantified both the short-term and long-term clinical efficacy of acupuncture for PSD. Our pooled data revealed that combining acupuncture with CSRT significantly improved short-term clinical effective rates, measured by the WST, and objective functional scores, indicated by the SSA, compared to CSRT alone. Crucially, our synthesized data provides promising evidence that these therapeutic gains are not merely transient. Meaningful clinical improvements were maintained at follow-up intervals of 3 months or more post-intervention. The sustained efficacy of the WST effective rate is supported by a moderate and highly consistent pool of studies (*k* = 5). More notably, the long-term data for objective functional assessment (SSA) has been successfully pooled (*k* = 2) and exhibited absolute statistical homogeneity (*I*^2^ = 0%), providing highly robust evidence for the cumulative and durable recovery of swallowing physiology. However, long-term data for subjective quality of life (SWAL-QOL, *k* = 2) remains relatively scarce. Furthermore, the absolute risk difference for adverse events was minimal (RD = 0.11), restricted entirely to mild, self-limiting local reactions, confirming a highly favorable overall safety profile.

### 4.2 Clinical interpretation of the efficacy outcomes

The clinical significance of our findings warrants careful interpretation across the diverse evaluation metrics. For the WST, the outcome was analyzed as a dichotomous clinical effective rate. By strictly defining “effective” as patients recovering to Grade I or II (representing safe swallowing with no or minimal aspiration risk), this metric inherently captures a highly meaningful clinical transition from dysphagia to functional recovery.

Regarding the objective SSA outcomes, the scoring system ranges from 18 to 46 points. According to recent diagnostic criteria (42), a score of ≥26 serves as the definitive cut-off for abnormal swallowing function (dysphagia). Consequently, the interval between the onset of clinical dysphagia (26 points) and a completely normal swallowing status (18 points) encompasses a remarkably narrow margin of just 8 points. Interpreting absolute score changes within this tight clinical window requires careful methodological consideration. While a universally established minimally clinically important difference (MCID) for the SSA remains absent in the current literature (43), recent clinical trials have empirically adopted an absolute score reduction of ≥4 points as a reliable indicator of meaningful clinical efficacy (44). In our meta-analysis, acupuncture interventions yielded an absolute SSA score reduction ranging from 3.3 to 5 points. This substantial decrease closely approaches or exceeds the empirical MCID threshold of 4 points, effectively bridging more than half of the diagnostic gap toward complete normality. Furthermore, this absolute functional improvement is robustly corroborated by our standardized mean difference analysis, which demonstrated a large effect size (SMD = −0.84) according to Cohen’s criteria.

Similarly, for the evaluation of patients’ subjective quality of life, previous literature has established a 6-point difference as a clinically meaningful threshold (MCID) for the SWAL-QOL measure (45). Our synthesized results demonstrated that both short-term (MD = 21.58) and long-term (MD = 23.56) improvements far surpassed this benchmark. Collectively, these multidimensional findings consistently confirm that the observed statistical improvements translate into substantial and highly relevant clinical benefits for patients with post-stroke dysphagia.

### 4.3 Neurobiological mechanisms underlying sustained efficacy

Unlike previous meta-analyses that focused exclusively on immediate post-treatment outcomes (9, 10), our analysis yielded two critical findings that warrant neurobiological explanation: particularly the sustained cross-period efficacy at follow-ups (e.g., the robust long-term SSA results with 0% heterogeneity). Furthermore, while both electroacupuncture and manual acupuncture demonstrated significant clinical efficacy, exploring the specific neurobiological pathways of these distinct stimulation modalities remains essential.

Basic research specifically elucidates the superiority of electroacupuncture: its consistent, quantifiable, and rhythmic low-frequency stimulation has been reported to robustly activate neural projections from the primary motor cortex (M1) to the nucleus tractus solitarii (NTS)—a pathway essential for the central coordination of the swallowing reflex (8). Additionally, targeted electroacupuncture stimulation is associated with the pronounced upregulation of brain-derived neurotrophic factor (BDNF) and promoted angiogenesis in ischemic regions (7, 46). By driving this central neural reorganization, electroacupuncture likely addresses the root neuropathology of dysphagia more efficiently than manual stimulation.

Furthermore, to explain why these clinical benefits do not rapidly decay upon treatment cessation, we must look beyond transient peripheral muscle activation to long-term structural plasticity (6). Translating preclinical findings to human subjects, emerging neuroimaging evidence offers a potential structural basis for our long-term results. Functional magnetic resonance imaging (fMRI) studies have demonstrated that acupuncture induces task-related cortical reorganization, enhancing functional connectivity within the swallowing motor network (e.g., bilateral primary sensorimotor cortex, insula) (47). More importantly, compelling evidence from diffusion tensor imaging (DTI) indicates that prolonged acupuncture intervention may facilitate the microstructural repair and integrity of descending white matter pathways, particularly the corticobulbar tract (CBT) (48). It is hypothesized that this deep-seated, durable structural repair within the central nervous system may contribute to the sustained, non-decaying functional recovery (such as the persistent reduction in SSA scores) observed in our patients months after the intervention ended. However, given the low-to-very-low certainty of the current long-term clinical evidence, these mechanistic links remain speculative and warrant further investigation.

### 4.4 Clinical implications of intervention timing and modality

A pivotal finding from our subgroup analysis is the nuanced impact of intervention timing. Interestingly, the relative risk for the short-term WST effective rate was notably higher when acupuncture was initiated during the subacute and recovery phases (>14 days to 6 months, RR = 1.57) compared to the acute phase (≤14 days, RR = 1.18). While the first 2 weeks post-stroke strictly designated as the early management phase by the AHA/ASA guidelines (41) are widely recognized as the golden window for neuroplasticity (49), the massive spontaneous neurological recovery occurring in the acute phase may statistically dilute the specific, observable add-on effect of acupuncture in clinical trials (4, 50). Conversely, during the subacute and recovery phases—when spontaneous recovery decelerates and conventional training yields diminishing returns—the neuromodulatory push provided by acupuncture becomes clinically profound (3).

Furthermore, regarding the choice of acupuncture modality, our subgroup interaction tests revealed nuanced results. While electroacupuncture showed a marginal potential advantage for the WST (*p* = 0.08), no statistically significant superiority was established for the objective SSA scores (*p* = 0.56). Both modalities effectively improved swallowing function. While electroacupuncture delivers consistent, quantifiable, and rhythmic low-frequency stimulation that may better facilitate the synchronization of pharyngeal muscle groups (51), current statistical evidence does not support exclusively prioritizing one modality over the other. The choice of stimulation should remain individualized based on clinical context.

### 4.5 Strengths and limitations

This study provides robust quantitative evidence for the full-cycle management of PSD by isolating studies with extended follow-up data. However, several methodological limitations must be thoughtfully considered. First, the inherent physical nature of acupuncture renders double-blinding hardly feasible, introducing an unavoidable potential for performance bias (52, 53).

Second, significant statistical heterogeneity was observed in specific outcome measures, particularly the short-term SSA score (*I*^2^ = 82%) and SWAL-QOL score (*I*^2^ = 79%). Our sensitivity analysis identified specific trials, such as Bai et al. (27), Xia et al. (34), and Kang et al. (22), as primary drivers of this heterogeneity. As discussed, these studies represent methodological and clinical extremes within our pool, ranging from isolated single-acupoint stimulation to distinct theoretical frameworks (e.g., Tongdu Tiaoshen) and prolonged or ultra-short treatment durations. This variation is likely multifactorial, deeply rooted in the inherent complexities of standardizing traditional East Asian medicine interventions (54). Across the included RCTs, clinical heterogeneity naturally stems from diverse acupoint selections, manual manipulation techniques, and varying treatment frequencies. Methodological discrepancies in control group designs also introduce substantial variance. For example, Xia et al. (34) rigorously employed a non-acupoint superficial needling sham control, whereas most other trials lacked any sham intervention. Furthermore, our subgroup analysis identified the timing of intervention (acute versus subacute/recovery phases) as a core source of heterogeneity for WST efficacy, highlighting a critical need to develop stage-specific treatment protocols in future research.

Third, a striking discrepancy regarding publication bias was observed between different outcome measures. While the asymmetrical funnel plot and Egger’s test (*p* < 0.001) confirmed significant publication bias for the short-term WST effective rate, no such bias was detected for the short-term SSA score (Egger’s test, *p* = 0.250). This indicates that the dichotomous, semi-subjective WST data is highly susceptible to small-study effects. Crucially, we recognize that this significant publication bias in the WST outcome is directly driven by two specific methodological design choices in our review. First, to ensure baseline methodological quality, we applied strict *a priori* database filters to restrict Chinese-language literature exclusively to CSCD and Peking University Core journals. While defensible for quality control, this upfront restriction structurally excluded a vast but unquantifiable volume of studies published in lower-tier journals, which are often more likely to report smaller or negative effect sizes. Second, our search strategy prioritized peer-reviewed publications with complete extractable datasets to ensure data reliability. Although our search of the Cochrane Library inherently captured clinical trial registry protocols (e.g., from ClinicalTrials.gov and ICTRP), they were strictly excluded because unpublished registry entries lack the comprehensive statistical parameters (e.g., standard deviations, exact event rates) required for robust quantitative synthesis and have not undergone rigorous peer review. However, we acknowledge that this methodological trade-off inevitably means registered but unpublished trials with negative results were not captured. Consequently, the WST effect sizes might be overestimated. Conversely, the continuous, objective nature of the SSA metric proved to be statistically robust and immune to such bias, reinforcing the reliability of our findings regarding functional recovery.

Furthermore, regarding our search methodology, because the Cochrane Library records were initially screened manually without an automated export log, the detailed screening breakdown was reconstructed retrospectively utilizing a specific date filter (up to March 18, 2026). We acknowledge this as a methodological limitation, as retrospective, filter-restricted database exports are not necessarily identical to contemporaneous searches, since record metadata (e.g., publication type, indexing) can be revised by the database provider after initial listing.

Finally, there is a pronounced clinical mismatch in the existing literature regarding long-term follow-up metrics. While an increasing number of trials are reporting long-term objective physiological data (such as SSA scores and nutritional indicators), there is a severe dearth of long-term patient-reported outcome measures (PROMs) evaluating subjective quality of life (e.g., SWAL-QOL). Future large-scale, multicenter trials utilizing standardized acupuncture protocols must bridge this gap by simultaneously incorporating objective physiological metrics (e.g., videofluoroscopic swallowing studies) and long-term psychological/social assessments to comprehensively validate the full-spectrum benefits of acupuncture.

## 5 Conclusion

Preliminary evidence suggests that acupuncture, when combined with conventional swallowing rehabilitation, serves as a potentially beneficial and safe adjunctive therapy for post-stroke dysphagia. It demonstrates a capacity to enhance both short- and long-term swallowing function. However, the current certainty of evidence for short-term efficacy is graded as low to moderate. Crucially, the evidence supporting the sustained long-term efficacy is also limited, graded as low for both WST and SSA, and very low for SWAL-QOL, primarily driven by high risks of bias, suspected publication bias, and imprecision stemming from small sample sizes in the long-term follow-up pools, rather than inconsistency in the direction of the effect. Future rigorously designed, large-scale, and sham-controlled RCTs are imperative to substantiate these findings and firmly establish its clinical and long-term utility.

## Data Availability

The original contributions presented in the study are included in the article/Supplementary material, further inquiries can be directed to the corresponding authors.
