# Supplementary material for: Sustained efficacy of acupuncture in post-stroke dysphagia: a systematic review and meta-analysis
Source: Front Neurol. 2026 Sep 21;17:1889347. doi: 10.3389/fneur.2026.1889347 (PMC13635353; doi:10.3389/fneur.2026.1889347)
Supplement: Supplementary file 2 [file Data_Sheet_2.docx]

Supplement 2: Detailed search strategies for each database

Search Period: From database inception to March 2026.

Part 1: English Databases

1. PubMed

Plaintext

((Stroke[MeSH Terms] OR "Post-Stroke"[Title/Abstract])) AND (("Deglutition Disorders"[MeSH Terms] OR "Dysphagia"[Title/Abstract] OR "Swallowing Disorders"[Title/Abstract])) AND (("Acupuncture"[MeSH Terms] OR "Acupuncture Therapy"[Title/Abstract] OR "Electroacupuncture"[Title/Abstract]))

2. Embase

Plaintext

(stroke OR poststroke) AND (dysphagia OR 'swallowing disorder') AND acupuncture

3. Cochrane Library

Plaintext

(acupuncture OR electroacupuncture OR "needle acupuncture" OR "scalp acupuncture" OR "body acupuncture" OR "neck acupuncture" OR "tongue acupuncture" OR "point acupuncture" OR "meridian acupuncture" OR "deqi acupuncture" OR "sham acupuncture") AND (stroke OR "cerebral stroke" OR "cerebrovascular accident" OR "CVA" OR "brain infarction" OR "cerebral infarction" OR "ischemic stroke" OR "hemorrhagic stroke" OR "cerebral hemorrhage" OR "intracerebral hemorrhage" OR "apoplexy") AND (dysphagia OR "swallowing disorder" OR "swallowing difficulty" OR "post-stroke dysphagia" OR "PSD" OR "oropharyngeal dysphagia" OR "pharyngeal dysphagia" OR "dysphagia, post-stroke") AND (randomized controlled trial OR RCT OR "randomized trials" OR "controlled clinical trial" OR "parallel group trial" OR "random allocation")

4. Web of Science (WOS) Core Collection

Plaintext

("Stroke*" OR "Cerebrovascular Accident*" OR "CVA*") AND ("Deglutition Disorder*" OR "Dysphagia" OR "Swallowing Disorder*" OR "Oropharyngeal Dysphagia") AND ("Acupuncture" OR "Electroacupuncture" OR "Needling" OR "Scalp Acupuncture")

Part 2: Chinese Databases

5. China National Knowledge Infrastructure (CNKI)

Plaintext

(SU='脑卒中' OR SU='中风' OR SU='脑梗死' OR SU='脑出血' OR SU='脑血管意外') AND (SU='吞咽障碍' OR SU='吞咽困难') AND (SU='针刺' OR SU='针灸' OR SU='电针' OR SU='项针' OR SU='舌针' OR SU='头针')

6. Wanfang Data

Plaintext

(主题:("脑卒中" OR "中风" OR "脑梗死" OR "脑出血" OR "脑血管意外")) AND (主题:("吞咽障碍" OR "吞咽困难" OR "球麻痹" OR "假性球麻痹")) AND (主题:("针刺" OR "针灸" OR "电针" OR "项针" OR "舌针" OR "头针"))

7. VIP Database for Chinese Technical Periodicals (VIP)

Plaintext

(M=(中风 OR 卒中 OR 脑卒中 OR 中风后)) AND (M=(吞咽障碍 OR 吞咽困难 OR 吞咽紊乱 OR 吞咽功能障碍)) AND (M=(针灸 OR 针刺疗法 OR 电针 OR 头针 OR 耳针))

8. Chinese Biomedical Literature Database (SinoMed / CBM)

Plaintext

("针刺"[全部字段] OR "Acupuncture"[全部字段] OR "药物针刺"[全部字段] OR "针刺"[主题词]) AND ("脑卒中"[全部字段] OR "卒中"[全部字段] OR "Stroke"[全部字段] OR "脑血管意外"[全部字段] OR "脑中风"[全部字段] OR "CVA(脑血管意外)"[全部字段] OR "CVAs(脑血管意外)"[全部字段] OR "脑血管中风"[全部字段] OR "急性中风"[全部字段] OR "急性卒中"[全部字段] OR "急性脑血管意外"[全部字段] OR "急性脑卒中"[全部字段] OR "卒中"[主题词]) AND ("吞咽障碍"[全部字段] OR "Deglutition Disorders"[全部字段] OR "吞咽困难"[全部字段] OR "口咽性咽下困难"[全部字段] OR "食管咽下困难"[全部字段] OR "咽下障碍"[全部字段] OR "吞咽障碍"[主题词]) AND ("随机对照试验"[全部字段] OR "Randomized Controlled Trial"[全部字段] OR "随机对照试验"[主题词])
